# Comparison of early visual quality in patients with moderate myopia using different optical zones in small incision lenticule extraction (SMILE)

**DOI:** 10.1186/s12886-020-01798-y

**Published:** 2021-01-19

**Authors:** Yan Wu, Zhenping Huang

**Affiliations:** Department of Ophthalmology, Jinling Hospital, No.305 East Zhongshan Road, Nanjing, 210002 Jiangsu Province People’s Republic of China

**Keywords:** SMILE, Moderate myopia, Optical zone, Visual quality

## Abstract

**Background:**

The early visual qualities of patients with moderate myopia were evaluated after small incision lenticule extraction (SMILE) using different optical zones.

**Methods:**

In this retrospective case study, 27 cases (51 eyes) were selected, including 10 cases in Group A (19 eyes), 6.6–6.8 mm in the optical zone, 10 cases in Group B (19 eyes), 6.4–6.5 mm in the optical zone, and 7 cases in Group C (13 eyes),6.1–6.3 mm in the optical zone. The following items were examined preoperatively and 1 month postoperatively: uncorrected visual acuity (UCVA), best-corrected visual acuity (BCVA), spherical, cylinder, central corneal thickness (CCT), corneal mean curvature (CMC), total ocular aberrations (TA), total low order aberrations (tLOAs), defocus, astigmatism and total high order aberrations (tHOAs), spherical, coma, trefoil, modulation transfer function (MTF), MTF_cutoff_, SR, objective scatter index (OSI), point scatter function at 50 and 10% (PSF50%, PSF10%), and contrast visual acuity of 100, 20, and 9% (VA100%, VA20%, and VA9%). We compared the three groups by Kruskal-Wallis test. Wilcoxon signed ranks test was used for each group before and 1 month after surgeries. *P*< 0.05 was considered statistically significant.

**Results:**

There was no significant difference in UCVA, BCVA, CCT, cylinder, and CMC in three groups preoperatively and 1 month postoperatively (*P*> 0.05). Comparison of the aberrations of the three groups showed statistically significant difference only in TA, tLOA, defocus, astigmatism and SA preoperatively, and trefoil 1 month postoperatively(*P*< 0.05). The postoperative TA, tLOAs, defocus, astigmatism and trefoil of the three groups were lower than those before surgeries (*P*< 0.05). The postoperative tHOAs of Group B and C was lower than those before surgeries (*P*< 0.05). The MTF results showed that before surgeries, there were significant differences in three groups (*P*< 0.05) in spatial frequencies 5~15 cycles per degree (cpd), and no differences in 20~30 cpd(*P*> 0.05), while no difference were observed in all spatial frequencies postoperatively (*P*> 0.05). Comparing the preoperative and postoperative MTF values for each group, the results showed that there was a significant difference in Group C at 5~20 cpd after surgeries(*P*< 0.05). There was no significant difference in MTF_cutoff_, SR, OSI, PSF50%, PSF10%, VA100%, VA20%, and VA9% in the three groups preoperatively (*P*> 0.05). One month after surgeries, higher VA9% values were measured for Group C compared to Group A and B (*P* < 0.05). There was no significant difference in each group before and after surgeries (*P*> 0.05).

**Conclusion:**

SMILE could improve the visual qualities of patients with moderate myopia. Reducing the surgical optical zone will only affect night vision slightly.

## Background

Small incision lenticule extraction (SMILE) was introduced in 2011 as an intrastromal technique for correction of myopia and myopic astigmatis m[1, 2]. Several studies have reported high efficacy, predictability, stability, and safety after SMIL E[3–10] with reduced risk of postoperative dry eye symptoms due to the flap-free techniqu e[11–13].

Although SMILE is commonly used for correcting myopic astigmatism, few prospective studies have evaluated the effect that the optical zone in SMILE has on postoperative visual quality. The purpose of this study was to compare the changes in early postoperative visual quality in different optical zones.

## Methods

### Patients

In this retrospective case study, 26 patients (51 eyes) who underwent SMILE surgery at the Optometry Center of Jinling Hospital from June to September in 2018 were selected. The inclusion criteria were: (1) 18–35 years old; (2) Preoperative spherical − 3 ~ − 6 diopters (D), cylinder 0 ~ − 2 D, equivalent spherical − 3 ~ − 6 D; (3) BCVA ≥ 0.8; (4) pupil diameter after dark adaptation ≥ 5.5 mm. The exclusion criteria were: (1) a history of eye surgery; (2) suffering from keratoconus or suspicious keratoconus; (3) diabetes, autoimmune diseases, and other systemic diseases; and (4) other eye diseases that affect visual function. For patients wearing contact lenses, the wearing of soft lenses was stopped for 2 weeks prior to surgery, and rigid lenses for 4 weeks.

### Surgery

All surgical procedures were performed by the same physician. We used the VisuMax femtosecond laser system. A 2–3 mm incision was made in the superior temporal cornea. The intrastromal lens thickness was calculated based on the corrected refractive power. The lens diameter (optical zone) was 6.1–6.8 mm, the cap thickness was 110–120 μm, and the cap diameter was 1.0 mm larger than the lens diameter. We first cut the substrate, bluntly separated and dissociated the lens through the incision, and finally removed the lens. Finally, we rinsed the corneal layers with balanced salt solution to remove the debris.

### Grouping

According to the optical zone used in the surgeries, all patients were divided into three groups: the optical zone was 6.6–6.8 mm in Group A (10 patients, 19 eyes), 6.4–6.5 mm in Group B (10 patients, 19 eyes) and 6.1–6.3 mm in Group C (7 patients, 13 eyes). The average age was (21.32±2.35) in Group A, (20.13±1.96) in Group B, and (21.43±2.01) in Group C. There was no significant difference among the groups (*P*> 0.05). After surgeries, all patients used 0.1% fluorometholone eye drops and 0.3% levofloxacin eye drops 4 times a day for 1 month (provided by Santen Pharmaceutical Co., China).

### Vision

The following parameters were measured preoperatively and 1 month after surgery for all patients: the uncorrected visual acuity (UCVA), the best-corrected visual acuity (BCVA), spherical value, cylinder value, mean corneal curvature (CMC), and central corneal thickness (CCT). We used corneal EyeSys corneal topography (provided by EyeSys Vision) to determine the CMC. UCVA and BCVA are expressed as logMAR values.

### Aberrations and modulation transfer function (MTF)

Using the iTrace visual function analyzer (manufactured by Tracey Co., USA), we measured the total aberrations (TA), total low order aberrations (tLOAs), defocus, astigmatism, total high order aberrations (tHOAs), spherical aberrations (SAs), coma, and trefoil, expressed as the root mean square (RMS). At the same time, we also measured the MTFs. All subjects underwent dark adaptation more than 30 min before the examinations, with pupil diameters > 5.5 mm. In Group A, the average pupil diameter was (6.31 ± 1.01) mm, (6.03±0.45) mm in Group B and (5.82 ± 0.73) mm in Group C. There was no significant difference among the groups (*P*> 0.05). We used the analyzer’s software (version 3.1) to unify the RMS values of 5.0 mm pupil diameter, to facilitate the expression and comparison.

### OQAS values

We measured the scattering index and objective visual quality with the double-pass OQAS-II system (Visiometrics Co., Spain), with a pupil diameter > 4 mm. The system showed the MTF cut-off frequency (MTF_cutoff_), Strehl ratio (SR), objective scatter index (OSI), point scatter function at 50 and 10% (PSF50%, PSF10%), and visual acuity at 100, 20, and 9% (VA100%, VA20%, VA9%). The VA is expressed in terms of logMAR.

### Statistical analysis

We used SPSS 21.0 software for analyzing the data, expressed by ($\overline{\mathrm{x}}\pm s$). Comparisons were made between the two groups using Kruskal-Wallis test. The preoperative and postoperative comparisons of each group were performed using Wilcoxon signed ranks test. *P*< 0.05 was defined as statistically significant.

## Results

### VA, refractive status, CMC, and CCT

**Fig. 1:**
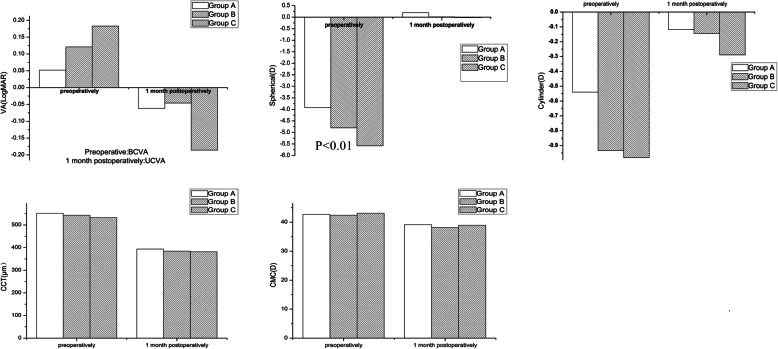
visual acuity (VA), refractive status, corneal mean curvature (CMC) and central corneal thickness (CCT) of three groups before and 1 month after the operations

The UCVA/BCVA, cylinder, CMC, and CCT values were not significantly different among the three groups before surgeries and 1 month after surgeries (*P*> 0.05). We compared the BCVA values of the three groups before surgeries, and UCVA values 1 month after surgeries. The spherical values were significantly different among the three groups before surgeries(*P*< 0.01), and not significantly different after surgeries. (Fig. 1).

### Ocular aberrations

**Fig. 2:**
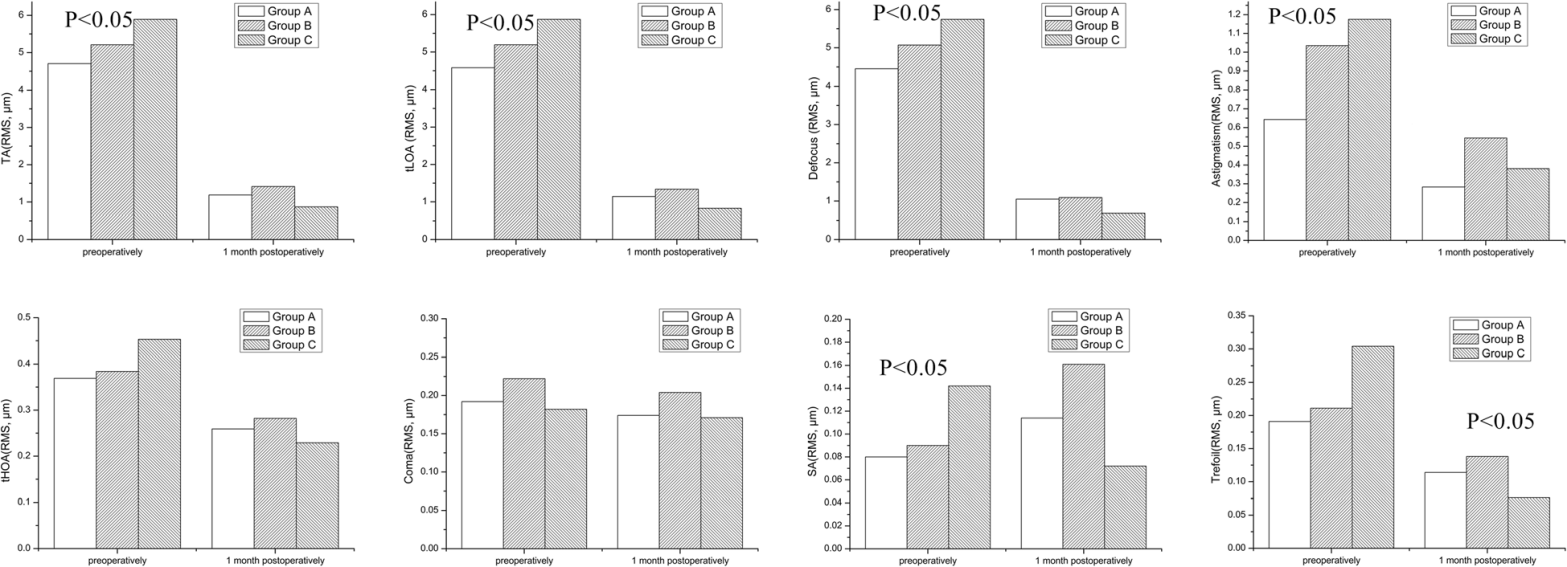
All levels of ocular aberrations of three groups before and 1 month after the operations. Ocular aberrations include total absorrations (TA), total low order aberrations (tLOAs), total high order aberrations (tHOAs), spherical aberration (SA), coma, and trefoil

The results showed that there were no significant differences in tHOAs, trefoil and coma before surgeries (*P*> 0.05), while TA, tLOAs, defocus, astigmatism and SA values were significantly different in three groups (*P*< 0.05). We measured the aberrations again 1 month after surgeries. There were significant differences in trefoil values in three groups (*P*< 0.05), while TA, tLOAs, defocus, astigmatism, tHOAs, coma, and SA exhibited no significant difference (*P*> 0.05). (Fig. 2).

### MTF

**Fig. 3:**
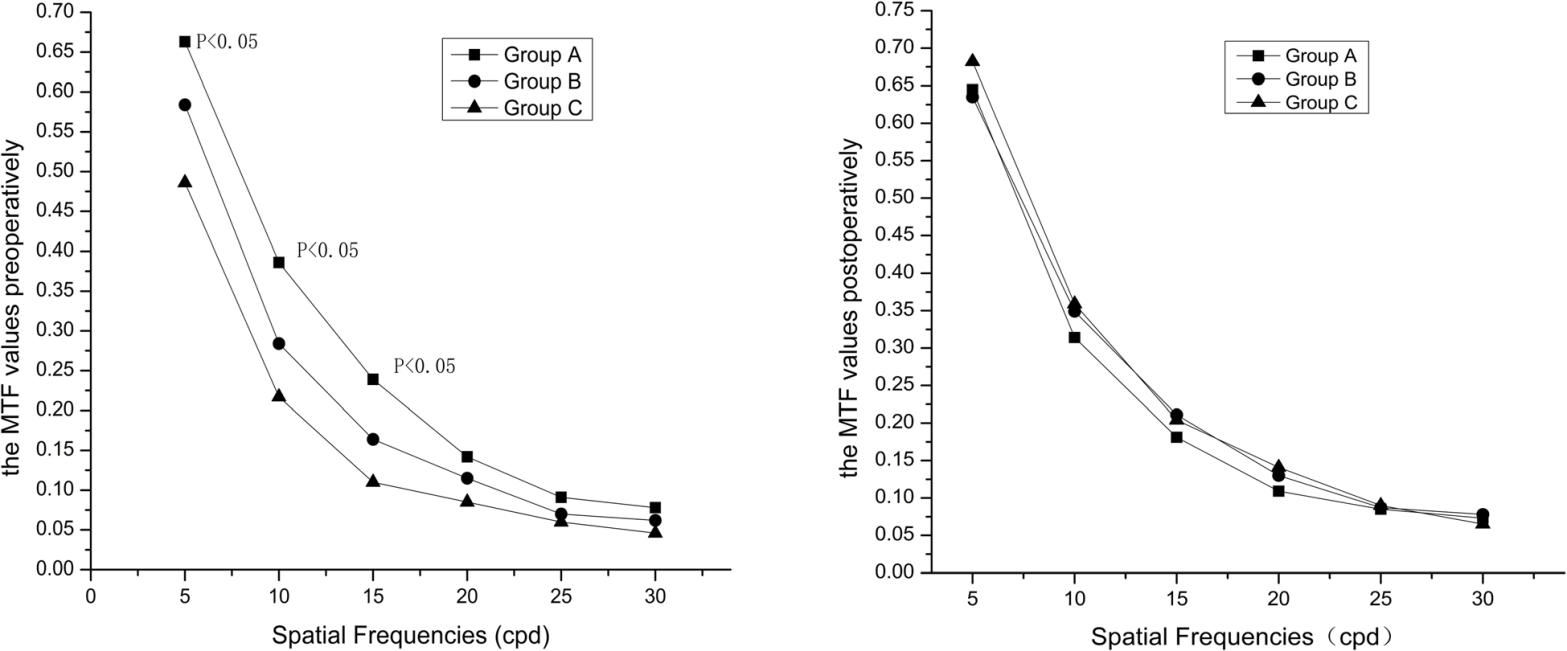
The modulation transfer function (MTF) values of three groups before and 1 month after the operations

We used iTrace to measure the MTF values at each spatial frequency before surgery. The results showed that before surgeries, there were significant differences in three groups (*P*< 0.05) in spatial frequencies 5~15 cycles per degree (cpd), and no differences in 20~30 cpd(*P*> 0.05). There was no significant difference in three groups in all spatial frequencies 1 month after surgeries (*P*> 0.05) (Fig. 3).

### OQAS values

**Fig. 4:**
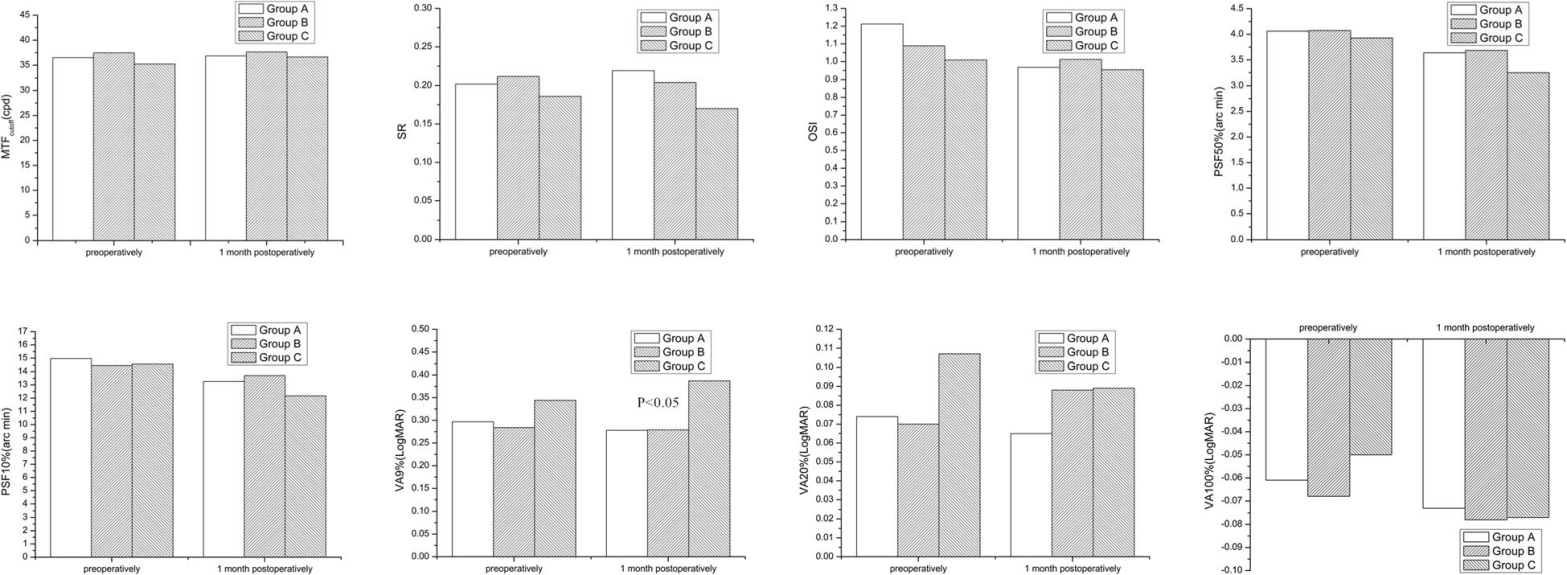
The OQAS values of three groups before and 1 month after the operations. The OQAS values include the MTF cut-off frequency (MTF_cutoff_), Strehl ratio (SR), objective scatter index (OSI), point scatter function at 50 and 10% (PSF50%, PSF10%), and visual acuity at 100, 20, and 9% (VA100%, VA20%, VA9%)

Before surgeries, the MTF_cutoff_, SR, OSI, PSF50%, PSF10%, VA100%, VA20% and VA9% showed no significant difference among the three groups(*P*> 0.05). 1 month after surgeries, MTF_cutoff_, OSI, SR, PSF50%, PSF10%, VA100% and VA20% values had no significant difference (*P*> 0.05), whereas VA9% had statistically significant (*P* < 0.05). The VA9% values of Group C were higher than those of Group A and B. (Fig. 4).

### Comparison of the aberration, MTF and OQAS values before and after surgeries

We analyzed the changes in aberrations, MTF and OQAS values in each group before and after surgeries. In Group A, B and C, the postoperative TA, tLOA, defocus, astigmatism and trefoil exhibited statistical differences compared with preoperative values (*P*< 0.05). The postoperative tHOA in Group B and C were significantly different from those before surgeries (*P*< 0.05), while there was no difference in Group A (*P*> 0.05). There was no significant difference in the changes in coma and SA in all groups(*P*> 0.05). The MTF values of Group C at 5–20 cpd 1 month after surgeries were significantly higher than those before surgeries (*P*< 0.05), while there was no significant difference at 25–30 cpd (*P*> 0.05). There was no significant difference in MTF values of all the spatial frequencies in Group A and B before and after surgeries (*P*> 0.05). There was no significant difference in MTF_cutoff_, OSI, SR, PSF50%, PSF10%, VA100%, VA20%, and VA9% for the three groups before and after surgeries (*P*> 0.05) (Table 1).

**Table 1:** The changes of the Aberrations and OQAS Values before and 1 month after the operations

	Group A				Group B				Goup C			
	preoperatively	postoperatively	Z	*P*	preoperatively	postoperatively	Z	*P*	preoperatively	postoperatively	Z	*P*
TA (μm)	4.704±0.907	1.117±0.775	−3.823	0.000	5.201±1.281	1.413±1.174	−3.680	0.000	5.891±0.925	0.868±0.403	−2.803	0.005
tLOAs (μm)	4.578±0.828	1.142±0.810	−3.823	0.000	5.194±1.279	1.337±1.134	−3.724	0.000	5.872±0.929	0.828±0.401	−2.803	0.005
defocus (μm)	4.451±0.824	1.048±0.857	−3.823	0.000	5.071±1.254	1.091±0.929	−3.724	0.000	5.742±0.937	0.681±0.404	−2.803	0.005
astigmatism (μm)	0.643±0.307	0.283±0.200	−3.220	0.001	1.035±0.520	0.544±0.504	−2.330	0.020	1.174±0.358	0.381±2.285	−2.803	0.005
tHOAs (μm)	0.369±0.199	0.259±0.080	−1.650	0.10	0.384±0.156	0.282±0.132	−2.156	0.031	0.453±0.124	0.230±0.134	−2.803	0.005
coma (μm)	0.192±0.140	0.174±0.104	−0.402	0.687	0.222±0.120	0.204±0.135	−0.436	0.663	0.182±0.092	0.171±0.141	−1.070	0.285
SA (μm)	0.080±0.049	0.114±0.075	−1.208	0.227	0.090±0.078	0.161±0.145	−1.502	0.133	0.142±0.068	0.072±0.063	−1.836	0.066
trefoil (μm)	0.191±0.126	0.114±0.054	2.377	0.046	0.211±0.117	0.138±0.069	−2.373	0.018	0.304±0.147	0.076±0.038	−2.803	0.005
MTF 5 cpd	0.685±0.200	0.645±0.153	−1.006	0.314	0.584±0.204	0.634±0.215	−0.828	0.408	0.486±0.228	0.682±0.158	−1.988	0.047
10 cpd	0.386±0.192	0.314±0.175	−1.368	0.171	0.284±0.193	0.349±0.224	−1.089	0.276	0.217±0.131	0.359±0.196	−1.988	0.047
15 cpd	0.239±0.137	0.181±0.120	−1.228	0.220	0.164±0.131	0.211±0.153	−0.719	0.472	0.110±0.064	0.204±0.137	−1.988	0.047
20 cpd	0.142±0.090	0.109±0.077	−1.248	0.212	0.115±0.083	0.130±0.105	−0.675	0.500	0.085±0.039	0.141±0.018	−1.988	0.047
25 cpd	0.091±0.071	0.085±0.060	−0.463	0.643	0.070±0.062	0.087±0.090	−1.677	0.094	0.061±0.035	0.091±0.056	−1.632	0.103
30 cpd	0.785±0.656	0.073±0.062	−0.322	0.747	0.062±0.057	0.079±0.086	−1.112	0.266	0.046±0.029	0.067±0.058	−0.102	0.919
MTF_cutoff_(cpd)	36.500±11.702	36.846±10.018	−0.414	0.679	37.463±12.056	13.700±6.465	−0.118	0.906	35.229±9.607	36.628±9.290	−0.089	0.929
SR	0.202±0.072	0.719±0.090	−0.672	0.501	0.212±0.074	0.204±0.064	− 0.118	0.906	0.186±0.052	0.170±0.052	−0.711	0.477
OSI	1.213±0.386	0.968±0.579	−1.766	0.077	1.089±0.752	1.011±0.683	−1.155	0.248	1.009±0.530	0.954±0.525	−0.822	0.824
PSF50%	4.060±1.852	3.638±1.200	−0.569	0.569	4.072±2.146	3.685±1.168	−0.639	0.523	3.926±1.012	3.248±0.909	−1.156	0.248
PSF10%	14.973±7.793	13.257±5.674	−0.724	0.469	14.468±8.702	13.700±6.465	−0.544	0.586	14.196±5.659	12.169±4.525	−0.356	0.722
VA100%(LogMar)	−0.061±0.150	−0.073±0.122	− 0.336	0.737	− 0.068±0.163	−0.078±0.132	− 0.283	0.777	− 0.050±0.123	−0.078±0.109	− 0.102	0.919
VA20%(LogMar)	0.074±0.174	0.065±0.160	−0.028	0.977	0.070±0.177	0.088±0.164	−0.142	0.887	0.107±0.137	0.089±0.144	−0.255	0.799
VA9%(LogMar)	0.297±0.173	0.278±0.182	−0.181	0.856	0.284±0.196	0.278±0.140	−0.281	0.779	0.344±0.130	0.410±0.115	−0.845	0.398

## Discussion

Prior to 2011, we performed refractive surgery usually by producing a corneal flap as used traditionally with LASIK and then ablating corneal stroma under the flap. After 2011, the SMILE separated a concave lens directly under the flap with a femtosecond laser and then removed the lens under a small incision. SMILE avoided opening the corneal flap, reduced the risk of corneal flap displacement and the damage to the subepithelial nerves, maintained the stability of the anterior corneal surface, and increased corneal biomechanical stabilit y[14]. The flap made by corneal stromal lenticule using femtosecond laser is more uniform, reducing postoperative astigmatism and coma aberratio n[15, 16]. Choosing the proper size of the optical zone is important for SMILE, which refers to the diameter of the cutting lens. We usually obtained a sufficiently large optical zone to maintain postoperative visual quality. However, when the patient’s corneal thickness is insufficient, we generally reduce the size of the optical zone to ensure the thickness of the peripheral cornea. Our question is whether reducing the optical zone would affect the postoperative visual quality. In this study, we reviewed the patients who received different optical zones in SMILE. We studied the changes of the early visual quality and ocular scatters of these patients.

We divided patients into three groups based on the size of the optical zone: Group A used a nomal optical zone (6.6–6.8 mm), Group B used a moderate reduced optical zone (6.4–6.5 mm), and Group C used a deeply reduced optical zone (6.1–6.3 mm). We analyzed the refractive status, CCT, corneal curvature, aberrations at all levels, MTF, MTF_cutoff_, OSI, SR, and VA before and 1 month after surgeries in the three groups.

We first focused on the patients’ refraction and cornea. The spherical, cylindrical, CCT and corneal curvatures of the three groups were significantly reduced after surgeries, which is normal after refractive surgeries. We compared the cylindrical, CCT, and corneal curvature values of the three groups before and 1 month after surgeries, which showed no statistical difference among the three groups. There was statistical difference of the spherical values among the three groups before surgeries and no statistical difference 1 month after suregeries.

Vision is only a subjective indicator of visual quality. The objective indicators for assessing visual quality were divided into two major categories: one based on the pupil plane, including RMS for describing aberrations, and the other based on the retinal plane, including PSF and MTF [17].

To evaluate the visual quality based on the pupil plane, we used the iTrace visual function analyzer to measure the patient’s TA and aberrations on all levels. iTrace used beam tracking technology to project many tiny laser beams onto the retina through the pupil plan e[18]. Each light projected 1 point on the retina. The position of the light spot in the retina and the dispersion were analyzed by the position detector. We obtained the low-order and high-order aberrations of the patients, and used the RMS calculated by the Zernike polynomial to quantitatively express the aberrations on all level s[19]. The aberrations obtained by iTrace visual function analyzer were objective visual quality indicators based on the pupil plane, and the results were subject to pupil size. Therefore, all patients required dark adaptation for at least 30 min before the examinations, with the pupil diameter > 5.5 mm. We compared the pupil diameters and found no statistical difference of the pupil diameter in three groups. For comparison convenience, we used iTrace 3.1 software to convert all pupil diameter aberrations to an RMS value of 5.0 mm.

We analyzed aberrations of three groups before and after surgeries. There were statistical differences in TA tLOA, defocus, astigmatism and SA in three groups before surgeries, and no statistical difference in other aberrations. The difference of the three groups 1 month after surgeries appeared only in trefoil. Our study found that the Group C had lower trefoil values. There was no statistical difference in other aberrations, including tLOA, defocus, astigmatism and SA. Pedersen’s study [20] found that SMILE could effectively treat astigmatism, with a small amount of undercorrection, similar to the results of our study. We compared the changes in aberrations before and after surgeries in each group, and found that postoperative TA, tLOAs and tHOA decreased. The main purpose of SMILE is to reduce tLOAs. The decrease in tHOAs was mainly attributed to trefoil. There was no statistical difference in the reduction of coma and SA. Ağca’s study [21] found that after SMILE, the SA, coma, and trefoil all decreased. Our study found that a statistical difference appeared only in the reduction of trefoil in all groups. In summary, we concluded that SMILE could reduce postoperative aberrations of patients, including not only tLOAs, but also trefoil. Decreasing the optical zone within a certain range did not significantly affect postoperative aberrations, especially tHOAs.

We used iTrace visual function analyzer to measure aberrations and MTF values. The iTrace system uses PSF to quantify the visual quality, and iTrace3.1 software to obtain the MTF curve through the Fourier transform, reflecting the ability of the optical system to transmit the different spatial frequency components of the object. MTF values describe the relationship between contrast at different frequencies and the image quality of optical systems. A large MTF value indicates a good visual qualit y[22]. The MTF curve shows a rapid decline from low to medium frequency, and the high frequency tends to zero. The quantitative and objective expression of the changes in the attenuation of human eye visual quality from low to high frequency is quantified and objectively expresse d[23]. In general, the high frequency reflects the details of the object, the intermediate frequency reflects the layers of the object, and the low frequency reflects the contour of the object. As a result, there was statistical difference in the MTF curve in three groups before surgeries in frequencies of 5~15 cpd and no difference in frequencies of 20~30 cpd.

We analyzed the reasons for the difference, probably because we preferred to choose patients with higher refractive power, greater astigmatism, or thinner corneas in the choice of small optical zone. In our study, the difference of the three groups was statistically significant in spherical values, and the cylindrical values of Group B and C were higher than those of Group A without statistical differences. The preoperative tLOAs, defocus and astigmatism values of Group C were higher than those of Group A with statistical difference, which also confirmed this point. However, the difference disappeared one month after surgeries. We compared the MTF values of each group before and after surgeries and found that there was a significant increase in the MTF values for Group C at low-to-medium frequencies (5–20 cpd), but no difference at high frequencies (25–30 cpd), while there was no difference in Group A and B at all frequencies. Our analysis indicated that the reason might be due to the bad vision quality before surgeries in Group C, which improved after surgeries. There was no statistical difference for postoperative MTF values among the three groups, demonstrating that reducing the diameter of the optical zone had no significant effect on the MTF values.

Additionally, we used the OQAS II objective visual analysis system to examine the visual quality of patients before and after surgery. The OQAS II system captures human eye retinal PSF images using dual-channel technology. There are 5 main indicators, described as follows. (1) MTF_cutoff_: MTF is obtained by Fourier transform from PSF, but the OQAS II system mainly uses MTF_cutoff_ to express visual quality, which refers to the spatial frequency corresponding to 0.01 MTF value as the cutoff frequency, reflecting the influence of scattering and aberration on visual image quality. (2) PSF 50 and 10%: This is the visual angle width of the 50 and 10% peak light intensity in the PSF cross-sectional image, and its unit is arc min. It reflects the approximate shape of the PSF image, and indirectly determines the effect of aberrations and scattering on the visual quality based on the PSF interface. (3) OSI: The PSF of human eyes can be divided into two regions. The light intensity of the small angle PSF is large, and that of the large angle PSF is small. Small-angle PSF reflects vision, contrast sensitivity, and aberrations. Large-angle PSF reflects scattering. OSI is the ratio of the light intensity of the PSF l2–20 arc min to the light intensity of the central peak, which mainly reflects the degree of the scattering of the human eye. (4) VA (100, 20, 9%) is the visual acuity at 100, 20, and 9% contrast and corresponds to daytime, evening, and night vision, respectively. Compared with subjective vision, this vision is only related to the optical system of the human eye and is not affected by the retina and nervous system. For convenience of comparison, we converted it to a logMAR value record. (5) SR refers to the ratio of the central light intensity of the imaging diffraction spot of the optical system with aberration and without aberration. A high SR value represents good visual qualit y[24].

Compared with the aberration values for RMS obtained by iTrace, the objective indicators for visual quality obtained by OQAS II are based on the plane of the retina, which was not affected by the size of the pupil. Comparing the OQAS values of the three groups, there were no statistical differences in OQAS values before surgeries and 1 month after surgeries. The postoperative differences among three groups were mainly in VA9%, and Group C had higher VA9% values than Group A and B. VA9% represents night vision, indicating that the reduction in the optical zone had no significant effect on daytime and evening vision, but may affect night vision. However, it must be recognized that in recent decades, the adequate light at night is common in the city, we rarely need to see objects at low contrast. Therefore, the importance of night vision is also reduced. But we should fulfill the duty of informing patients in order to reduce the impact on the patients’ postoperative lives, because night vision would be important in some specific occasions, for example, for driving people in rural areas. The changes in the OQAS values before and after surgeries for each group were not statistically different, demonstrating that the surgeries did not decrease the patients’ visual quality.

## Conclusion

In summary, we have concluded that SMILE could improve the visual qualities of patients with moderate myopia. SMILE not only corrected the patients’ refractive errors, but also reduced high-order aberrations. Reducing the surgical optical zone will only affect night vision slightly. It is safe to reduce the optical zone in surgeries if it is necessary for patients.

## Data Availability

The datasets used or analysed during the current study are available from the corresponding author on reasonable request.

## Abbreviations

SMILE: Small incision lenticule extraction
UCVA: The uncorrected visual acuity
BCVA: The best-corrected visual acuity
CMC: Mean corneal curvature
CCT: Central corneal thickness
MTF: Modulation transfer function
TA: Total absorrations
tLOAs: Total low order aberrations
tHOAs: Total high order aberrations
SA: Spherical aberration
MTF_cutoff_: Root mean square, RMS;MTF cut-off frequency
SR: Strehl ratio
OSI: Objective scatter index
PSF: Point scatter function
VA: Visual acuity
